# Transforming breast cancer management with real-world data and artificial intelligence

**DOI:** 10.1016/j.esmorw.2024.100067

**Published:** 2024-08-22

**Authors:** P. Heudel, B. Mery, H. Crochet, T. Bachelot, O. Tredan

**Affiliations:** 1 Medical Oncology Department, Centre Léon Bérard, Lyon; 2 Head of Data Factory, Centre Léon Bérard, Lyon, France

**Keywords:** breast cancer, real-world data, real-world evidence, big data, artificial intelligence, natural language processing

## Abstract

**Background:**

Real-world data (RWD) provide essential insights into the effectiveness and safety of breast cancer treatments, particularly in diverse patient populations, where traditional clinical trials may have limitations. Integrating RWD into breast cancer research enhances the understanding of treatment outcomes and supports clinical decision-making, complementing the findings from controlled clinical studies.

**Design:**

This article reviews the integration of RWD into breast cancer research, highlighting the benefits and challenges. Various sources of RWD, including electronic health records (EHRs), insurance claims, and patient registries, are examined, with a focus on their application in studies of triple-negative breast cancer. The article also explores the role of artificial intelligence (AI) in managing RWD, particularly through technologies like natural language processing (NLP) and predictive analytics, which enhance data collection, storage, and analysis.

**Results:**

RWD has demonstrated significant value in informing clinical decision-making and improving patient outcomes in breast cancer treatment. The integration of AI into the management of RWD has provided deeper insights into patient outcomes and supported personalized treatment strategies. Specific studies leveraging RWD have shown improved understanding of breast cancer subtypes, such as triple-negative breast cancer, and enhanced the effectiveness of treatment protocols.

**Conclusion:**

Despite the benefits, challenges remain in integrating RWD and AI into clinical practice, particularly regarding transparency, interpretability, and ethical considerations. Addressing these challenges requires robust data governance frameworks, interdisciplinary collaboration, and investment in advanced analytical tools. The potential for RWD and AI to transform breast cancer treatment and improve patient care is significant, underscoring the need for ongoing research and collaboration.

## Highlights

• Uses RWD from EHRs, claims, and registries to improve breast cancer treatment.
• Explores AI like NLP and analytics in managing RWD for personalized treatment.
• Discusses RWD and AI integration, focusing on transparency, ethics, and governance.

## Introduction to RWD and their importance in breast cancer

Real-world data (RWD) are derived from sources outside of traditional clinical trials, such as electronic health records (EHRs), insurance claims, patient registries, and patient-reported outcomes (Table 1). These data provide valuable insights into the effectiveness and safety of treatments in broader and more diverse patient populations over extended periods.^1^^,^^2^ RWD complements traditional clinical research by filling in the gaps left by randomized controlled trials (RCTs). While RCTs are considered the gold standard for evaluating treatment efficacy, they often involve highly selective patient groups and controlled environments that may not reflect real-world settings. In contrast, RWD captures the complexity and variability of routine clinical practice, offering a more comprehensive understanding of treatment impacts.

**Table 1:** Examples of RWD sources

Source of RWD	Description	Examples
Electronic health records (EHRs)	Data collected during routine clinical care, including diagnoses, treatments, and outcomes.	EHRs from hospitals, clinics, physician offices.
Insurance claims	Information on treatments and medical services provided to patients, including costs and reimbursements.	Databases from health insurance companies.
Patient registries	Structured collections of data on patients with specific diseases, followed over long periods.	Cancer registries, national rare disease registries.
Patient-reported outcomes (PROs)	Data collected directly from patients regarding their health and quality of life.	Surveys, quality of life questionnaires, symptom tracking apps.
Wearable devices	Data generated by medical devices and connected health applications.	Glucose monitors, physical activity tracking apps, cardiac monitoring devices.
Observational studies	Data from non-interventional studies where treatments and outcomes are observed in real-world settings.	Cohort studies, case-control studies, post-marketing surveillance studies.
Pharmacy databases	Information on prescriptions and medication usage.	Pharmacy sales data, medication reimbursement databases.
National and international registries	Data collected by public health agencies for monitoring and surveillance purposes.	National cancer registries, international health registries.
Health surveys	Population health data collected through surveys and questionnaires.	National Health Interview Survey (NHIS), Behavioral Risk Factor Surveillance System (BRFSS).

For example, a study by Fredriksson et al.^3^ on triple-negative breast cancer (TNBC) demonstrated how RWD could provide critical insights into the effectiveness of treatments across various patient demographics and real-world settings. By analyzing data from a broad patient population, the study highlighted factors influencing treatment efficacy, thereby informing clinical decision making and potentially improving patient outcomes. Supporting this, Gyawali et al. highlighted the significance of RWD in understanding long-term outcomes and adverse effects in breast cancer treatments.^4^ This systematic review evaluated 43 studies on eHealth interventions for breast cancer supportive care, involving 6285 patients. While patients were generally satisfied with eHealth tools like mobile apps and online portals, the results for symptom and lifestyle improvements were inconsistent. The review highlights the need for further research focused on enhancing patient symptoms and addressing study heterogeneity and bias. Feinberg et al. utilized EHRs to identify patterns in treatment responses among diverse populations, providing essential data that can lead to personalized treatment strategies.^5^ Furthermore, Khozin et al. illustrated the use of insurance claims data to evaluate the real-world effectiveness of breast cancer therapies, uncovering discrepancies between clinical trial results and actual patient experiences.^6^ Another example of RWD sources is the study by Hershman et al., which analyzed patient registries to assess the comparative effectiveness of various treatments, contributing to more informed therapeutic choices.^7^ Additionally, a study by the Breast Cancer Centre in Berlin assessed the quality of life in breast cancer patients who underwent different surgical options using RWD. This study found significant differences in patient-reported outcomes between those undergoing breast-conserving surgery and mastectomy, emphasizing the importance of considering patient preferences and long-term quality of life in treatment planning.^8^

Controlled clinical trials often restrict the clinical and demographic characteristics of patients due to stringent inclusion and exclusion criteria, leading to the underrepresentation of various cancer patient profiles. Notably, real-world studies can provide insights into treatment patterns and the clinical effectiveness or safety in a diverse population during routine clinical practice, which can be valuable for clinical decision making.^9^, ^10^, ^11^, ^12^ For instance, in the case of palbociclib, RWD bolstered the clinical data for male breast cancer patients, resulting in a label expansion in 2019 to include men with hormone receptor-positive (HR+)/human epidermal growth factor receptor 2-negative metastatic breast cancer.^13^^,^^14^

However, a recent study aimed to explore how RWD are integrated into academic, investigator-initiated clinical research through an online survey distributed to cancer cooperative groups in Europe, North America, South America, Asia, and Oceania.^15^ A total of 125 cooperative groups from 58 countries, covering 13 cancer domains, participated. Although 67.2% lacked formal policies for RWD usage, 68.0% had previously conducted RWD studies for exploratory and confirmatory purposes. These groups primarily used observational RWD from disease registries, EHRs, and patient questionnaires, noting low costs and large-scale benefits but also facing methodological and operational challenges. There was no consensus on the definition of RWD. Despite experience with RWD, traditional clinical trials remain predominant; however, 62.5% of groups without prior RWD studies plan to initiate them. Cancer cooperative groups are incorporating RWD into their research, yet they face knowledge gaps and lack agreement on the definition of RWD, with conventional clinical trials still being prioritized.

These different examples underscore the vital role of RWD in enhancing our understanding of breast cancer treatments beyond the confines of controlled clinical trials, thereby contributing to improved patient care and outcomes.

## The impact of AI on managing RWD

Artificial intelligence (AI) has been around for a long time, but recent advancements in storage and computing capabilities have drastically transformed its potential. These developments have enhanced AI's ability to manage RWD, streamlining the collection, storage, and analysis of vast datasets. AI technologies now facilitate the efficient extraction and processing of data from various sources, enabling comprehensive and timely analyses.

### Facilitating data collection and storage

AI-driven systems improve the efficiency of data collection by automating the extraction of relevant information from diverse sources such as EHRs, laboratory reports, and insurance claims. This automation reduces the manual effort required and minimizes errors associated with data entry.^16^^,^^17^ Furthermore, AI enhances data storage by organizing and structuring large datasets in ways that make them more accessible and analyzable.^18^^,^^19^

For instance, an AI algorithm can be trained to recognize and extract specific data points from EHRs, such as patient demographics, treatment dates, and medication details.

Input data include the following:

• Raw EHR data containing patient records, laboratory results, and treatment histories.
• Unstructured clinical notes from health care providers.
• Insurance claims data including treatment costs and patient demographics.

Output data include the following:

• Structured datasets with extracted information on patient demographics, treatment timelines, and outcomes.
• Organized databases enabling easier access and analysis.

#### Example in breast cancer

An AI algorithm is deployed to extract and structure data from the EHRs of breast cancer patients. The algorithm identifies key variables such as tumor size, HR status, and treatment regimens. These structured data are then used to analyze treatment outcomes across different patient demographics, helping to identify patterns and improve clinical decision making.

### Enhancing data analysis

One of the most impactful applications of AI in RWD is natural language processing (NLP), which extracts meaningful information from unstructured clinical notes, pathology reports, and other text-based data.^20^^,^^21^ NLP algorithms can identify patterns and trends that traditional analysis methods might overlook, providing deeper insights into clinical practice. Large language models (LLMs), such as GPT-4, build on NLP capabilities to provide more sophisticated language understanding and generation. While NLP focuses on extracting specific information, LLMs can generate coherent text based on input data, making them useful for creating detailed patient summaries and generating predictive text-based models. NLP is primarily used for extracting and structuring information from human language, useful for tasks like summarizing clinical notes and extracting diagnostic information. LLMs can generate new content and provide context-based predictions, useful for creating detailed patient summaries and assisting in decision making by predicting outcomes based on historical data. By leveraging both NLP and LLMs, we can enhance the analysis of human language in administrative and diagnostic tasks while using predictive AI methods to forecast treatment outcomes and personalize care strategies.

### Foundational understanding of AI in RWD and clinical practice

**Figure 1:**
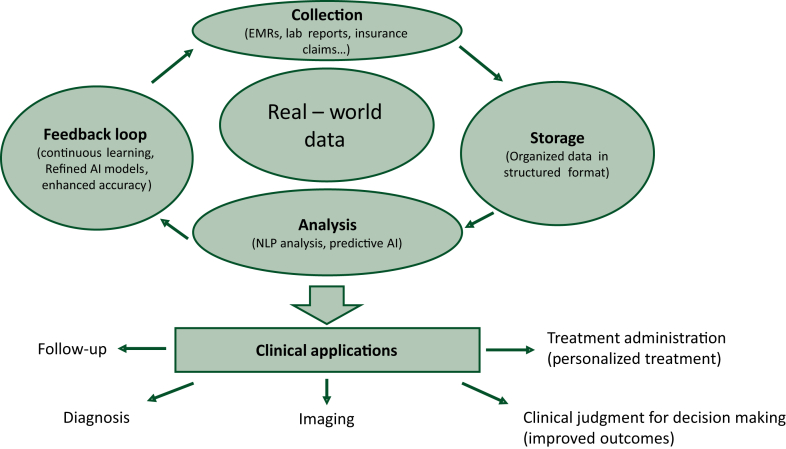
**Potential enhanced clinical workflow of breast cancer with AI and integration of RWD.** AI, artificial intelligence; EMR, electronic medical record; NLP, natural language processing; RWD, real-world data.

AI model development starts with data collection and preparation. Large volumes of data from EHRs, laboratory reports, and patient registries, are cleaned to ensure high-quality input. Next, the model is trained using algorithms such as decision trees or neural networks, learning from the data to identify patterns. Its performance is validated on a separate dataset to ensure generalizability. After training, the model is tested for accuracy and reliability, with fine-tuning adjustments made as needed. Validation involves cross-validation and external validation to confirm robustness and generalizability. AI enhances clinical decisions but does not replace clinical judgment. Clinicians must interpret AI insights within their expertise and patient-specific factors. AI models can inherit biases from training data, and poor-quality input can affect predictions. AI may not account for all contextual factors relevant to a patient’s condition. Consider an AI algorithm designed to predict breast cancer patient responses to chemotherapy. The algorithm collects data on age, tumor size, genetic markers, and treatment outcomes, learning to predict positive responses. For new patients, the algorithm analyzes their data to predict chemotherapy success. Predictions are validated with historical data to ensure accuracy, aiding clinicians in decision making while considering other clinical factors. This predictive capability supports the development of personalized treatment plans, enhancing the efficacy of interventions and reducing adverse effects. Additionally, AI tools assist in monitoring patient progress and adjusting treatments in real time, contributing to more responsive and adaptive health care delivery (Figure 1). Several case studies highlight AI’s potential in addressing specific clinical issues in breast cancer treatment. For instance, AI algorithms have been used to predict patient responses to chemotherapy by analyzing historical treatment data and genetic markers.^22^ These predictions enable clinicians to tailor treatments to individual patients, improving outcomes and minimizing unnecessary side-effects. Another example is the use of AI to analyze imaging data. This study investigates the use of machine learning (ML) to predict the histological response to neoadjuvant chemotherapy in early TNBC patients using whole-slide images and clinical data.^23^ To address data privacy and the biases of small-scale studies, the study employs federated learning across multiple centers. Results show that collaborative ML models improve prediction accuracy, rivaling expert-annotated approaches. This proof of concept highlights the potential of federated learning for biomarker discovery using large, real-world datasets.

### Importance of accurate analysis and interpretation

Accurate analysis and interpretation of RWD are vital for making informed clinical decisions.^24^ AI significantly enhances this process by ensuring data precision and uncovering subtle relationships within the data that might otherwise go unnoticed. The advanced analytical capabilities of AI can provide deeper insights into patient outcomes, treatment efficacy, and disease patterns. However, the integration of AI into clinical practice is not without challenges.^25^ One of the most critical aspects is ensuring that AI models are transparent and interpretable. Clinicians must be able to understand how these models arrive at their conclusions to trust and effectively use the insights they provide. This transparency allows for greater confidence in the AI-driven recommendations, ensuring that the insights are not only actionable but also reliable and aligned with clinical expertise.

Moreover, the integration of transparent AI models into clinical workflows facilitates a smoother adoption process. When clinicians can clearly see the decision-making process of AI tools, they are more likely to embrace these technologies and incorporate them into their daily practice. This acceptance is crucial for the successful implementation of AI in health care, as it ensures that the potential benefits of AI—such as improved patient outcomes and more efficient clinical processes—are fully realized. On the other hand, it is fundamental to keep in mind that AI can make mistakes. A recent study investigates the impact of incorrect AI results on radiologist performance and explores strategies to mitigate errors.^26^ Six radiologists interpreted chest radiographs with and without sham AI results, which included false positives and negatives. Findings revealed that incorrect AI increased both false-negative and false-positive rates compared to no AI assistance. However, errors were reduced when radiologists believed AI results would be deleted or when AI visually highlighted the area of interest. Despite potential benefits, reliance on AI can lead to significant diagnostic errors, underscoring the need for careful implementation and training. Ensuring the transparency and interpretability of AI models is essential for building clinician trust, integrating AI into clinical workflows, and ultimately providing actionable and reliable insights that enhance patient care.^27^

## Integration and future perspectives

Integrating RWD data and AI into clinical practice requires strategic planning and consideration of various challenges. Effective strategies include developing robust data governance frameworks, fostering interdisciplinary collaboration, and investing in advanced analytical tools.^28^^,^^29^

### Strategies for effective integration

1. Developing robust data governance frameworks: Establishing clear policies and procedures for data management, privacy, and security is essential to ensure the ethical use of RWD data. This includes compliance with regulatory standards and maintaining patient confidentiality.
2. Fostering interdisciplinary collaboration: Collaboration between data scientists, clinicians, and other health care professionals is crucial for leveraging the full potential of RWD and AI. Interdisciplinary teams can work together to identify relevant research questions, develop appropriate methodologies, and interpret findings in a clinically meaningful way.
3. Investing in advanced analytical tools: Health care organizations should invest in state-of-the-art analytical tools and platforms that can handle large volumes of data and carry out complex analyses. These tools should be user-friendly and integrate seamlessly with existing clinical systems.

### Addressing challenges and ethical considerations

The integration of RWD and AI into clinical practice also presents challenges, such as managing data privacy and addressing ethical concerns.^30^ Ensuring the confidentiality and security of patient data is paramount to maintaining patient trust and complying with legal requirements. Additionally, ethical considerations must be addressed, such as ensuring fairness in AI algorithms and preventing biases that could affect treatment decisions.

### Opportunities for improved patient care

The potential benefits of integrating RWD and AI into clinical practice are substantial.^31^^,^^32^ Enhanced data analytics can lead to more personalized and effective treatments, improving patient outcomes and optimizing resource allocation. Additionally, continuous learning from RWD can drive innovations in breast cancer treatment and contribute to the overall advancement of oncology care.

## Conclusion

The synergy between RWD data and AI holds significant promise for transforming breast cancer treatment. By leveraging these technologies, the health care community can achieve a deeper understanding of treatment effects, personalize care, and ultimately improve patient outcomes. As the field evolves, ongoing research and collaboration will be essential to overcome challenges and fully realize the potential of RWD and AI in oncology.
